# ﻿Identification of two new species of *Mecistocephalus* (Chilopoda, Geophilomorpha, Mecistocephalidae) from southern China and the re-description of *Mecistocephalussmithii* Pocock, 1895

**DOI:** 10.3897/zookeys.1218.130709

**Published:** 2024-11-13

**Authors:** Yang-Yang Pan, Jia-Bo Fan, Chun-Xue You, Chao Jiang

**Affiliations:** 1 Tianjin Key Laboratory of Agricultural Animal Breeding and Healthy Husbandry, College of Animal Science and Veterinary Medicine, Tianjin Agricultural University, Tianjin 300392, China National Resource Center for Chinese Materia Medica, China Academy of Chinese Medical Sciences Beijing China; 2 State Key Laboratory for Quality Ensurance and Sustainable Use of Dao-di Herbs, National Resource Center for Chinese Materia Medica, China Academy of Chinese Medical Sciences, Beijing 100700, China Tianjin Agricultural University Tianjin China

**Keywords:** COI, phylogeny, taxonomic key, taxonomy

## Abstract

*Mecistocephalus* Newport, 1843 is the most diverse genus in the family Mecistocephalidae; however, only two species have been recorded in mainland China to date. Therefore, taxonomic research on Chinese *Mecistocephalus* needs further research. In this study, the species diversity of *Mecistocephalus* in southern China was investigated using the mitochondrial marker COI integrated with morphological evidence. Species delimitation using Automatic Barcode Gap Discovery, Poisson Tree Processes, and phylogenetic and morphological analyses revealed ten species, including two newly described species, *M.chuensis* Jiang & You, **sp. nov.** and *M.huangi* Jiang & You, **sp. nov.** Furthermore, based on newly collected specimens, the presence of the little-known species *M.smithii* Pocock, 1895 was confirmed in China and thoroughly re-described.

## ﻿Introduction

*Mecistocephalus* Newport, 1843 is the most diverse genus in the family Mecistocephalidae Bollman, 1893, comprising nearly 70% of mecistocephalid species. Approximately 135 species in the genus have been reported to date, most of which are distributed in tropical and subtropical Asia, particularly in South Asia (India, Sri Lanka, and Nepal), Southeast Asia (Cambodia, Laos, Indonesia, Malaysia, Myanmar, Philippines, Singapore, Thailand, and Vietnam), and East Asia (southern China and Japan), with fewer records from Africa, America, and temperate areas, such as Northeast China (Bonato et al. 2011, 2016).

The earliest species discovered in China is *Mecistocephalussmithii* Pocock, 1895, reported in Ningbo, Zhejiang Province (Pocock 1895). Subsequently, taxonomic and faunistic contributions were made by F. Silvestri (1919), K.W. Verhoeff (1937), Y. Takakuwa (1940), and C.G. Attems (1947). A dozen *Mecistocephalus* species have been recorded in Taiwan and its adjacent regions. Wang and Mauries (1996) compiled a comprehensive record of 16 *Mecistocephalus* species from China. However, subsequent assessments by Uliana et al. (2007) identified four of these as synonyms (*M.fenestratus* Verhoeff, 1934 = *M.japonicus* Meinert, 1886; *M.insulomontanus* Gressitt, 1941 = *M.marmoratus* Verhoeff, 1934; *M.mirandus* Pocock, 1895 = *M.japonicus* Meinert, 1886; *M.takakuwai* Verhoeff, 1934 = *M.diversisternus* (Silvestri 1919)), and records of another two species in Taiwan were doubtful (*M.punctifrons* Newport, 1843 and *M.insularis* (Lucas, 1863)). Two species were misclassified: *Formosocephaluslongichilatus* (Takakuwa, 1936) = *M.longichilatus* Takakuwa, 1936 and *Taiwanellayanagiharai* Takakuwa, 1936 = *M.yanagiharai* (Takakuwa, 1936) (Bonato et al. 2002, 2003); accordingly, 14 *Mecistocephalus* species have been reported in China to date. Chao et al. (2020) have reported only the occurrence of *M.rubriceps* Wood, 1862 in Yunnan province; however, earlier research has primarily concentrated on Taiwan, and taxonomic knowledge of Chinese *Mecistocephalus* is incomplete.

In this study, we describe two new species of *Mecistocephalus* from southern China and re-describe *M.smithii*. Through a molecular analysis of the COI sequence, we verified the boundaries between the new species and their congeners.

## ﻿Materials and methods

### ﻿Specimen collection and identification

**Figure 1.**
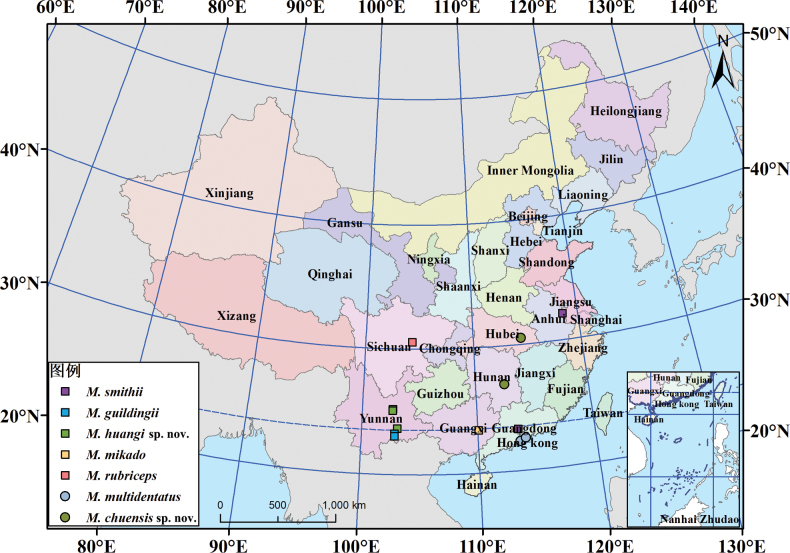
The known distribution of *Mecistocephalus* specimens in mainland China.

In China, 92 *Mecistocephalus* specimens were collected from seven provinces (Sichuan, Yunnan, Guangxi, Guangdong, Hunan, Hubei, and Jiangsu) (Fig. 1). All specimens were preserved in 75% ethanol. The forcipular segment was dissected and mounted for examination with lactic acid. Temporary mounts were clarified using lactic acid. The specimens are deposited in the National Resource Center for Chinese Materia Medica, China Academy of Chinese Medical Sciences, China (**CMMI**).

The terminology used in descriptions follows Bonato et al. (2010). Specimens were examined and photographed by using a Leica M205 FCA stereomicroscope and an Olympus BX51 Microscope. Line drawings were prepared from photographs taken with the microscope. The ArcMap 10.7.1 software tool was used to produce distribution maps.

### ﻿DNA extraction and fragment amplification

According to the DNeasy Blood & Tissue Kit (Qiagen, Hilden, Germany) requirements, ~ 6 walking legs from each specimen were used to extract genomic DNA; the isolated DNA being resuspended in 100 μL of buffer and stored at −20 °C for subsequent analysis. Polymerase chain reaction (PCR) was used to amplify the cytochrome c oxidase subunit I (COI). The PCR primers and programs used are listed in Table 1 (TFP682 and TRP682 are newly developed primers for more efficient amplification of *Mecistocephalus* CO1 sequences). PCR reaction mixtures (total volume 25 μL) contained 12.5 μL of 2 × M5 Mix (Transgen, Beijing, China), 0.4 μL each of the forward and reverse primers (10 μmol·L^-1^, Sangon, Shanghai, China), and 1 μL (~ 20 ng) of genomic DNA. Amplification was performed in a Veriti^TM^ thermal cycler (Applied Biosystems, Foster City, CA, USA) using cycling conditions in Table 1.

**Table 1.** Primers and PCR conditions employed.

Loci	Primer name	Sequence 5’– 3’	Program	Reference
CO1	LCO1490	GGTCAACAAATCATAAAGATATTGG	95 °C 5 min; 38 cycles of 20 s at 95 °C, 20 s at 45 °C and 1 min at 72 °C; 3 min at 72 °C	Folmer et al. 1994
	HCOUTOUT	GTAAATATATGRTGDGCTC		
CO1	LCO1490	GGTCAACAAATCATAAAGATATTGG	2 min at 94 °C; 35 cycles of 15 s at 95 °C, 40 s at 45–47 °C and 15 s at 72 °C; 10 min at 72 °C	Folmer et al. 1994; Joshi and Karanth 2011
	HCO2198	TAAACTTCAGGGTGACCAAAAAATCA		
CO1	TFP682	TTGGAGATGACCAAACATATAA	5 min at 94 °C, 30 s at 94 °C; 35 cycles of 30 s at 52 °C and 1 min at 72 °C; 5 min at 72 °C	This study
	TRP682	CAAAAAATCAGAATAGGTGTTG		

### ﻿Species delimitation, phylogenetic analysis, and genetic distance calculation

Species delimitation analyses were conducted using the Automatic Barcode Gap Discovery (ABGD) method (Puillandre et al. 2012) and the Poisson Tree Processes (PTP) model (Zhang et al. 2013) based on *COI* sequences. We ran ABGD under the following parameters: Pmin = 0.001, Pmax = 0.1, Steps = 20, X (relative gap width) = 1.0, Kimura (K80) TS/TV distances = 2.0, Nb bins (for distance distribution) = 20. We ran PTP under the following parameters: No. MCMC generations = 100000, Thinning = 100, Burn-in = 0.1, Seed = 123. The genetic distance between *Mecistocephalus* species was calculated using the Kimura 2-parameter model in MEGA 11 (Tamura et al. 2021). Using *Tygarrupjavanicus* (Attems, 1907) as an outgroup, the ClustalW tool (Thompson et al. 1994) in BIOEDIT 7.1.3.0 (Hall 1999) was used to align a total of 28 COI sequences obtained from 15 newly sequenced *Mecistocephalus* sequences and 13 sequences obtained from GenBank (12 sequences were *Mecistocephalus* and 1 sequence was *Tygarrup*) (Table 2). Phylogenetic trees based on Bayesian Inference (BI) and Maximum Likelihood (ML) were respectively constructed with the PHYLOSUITE 1.2.2 platform (Zhang et al. 2020). In addition, MODELFINDER (Kalyaanamoorthy et al. 2017) was used to select suitable models, in which the best ML model was TIM2+F+I+G4, and the best BI model was SYM+I+G4. IQ-TREE 1.6.8 (Nguyen et al. 2015) was used to perform ML analysis with 500,000 ultrafast bootstraps (Hoang et al. 2018). MRBAYES 3.2.6 (Ronquist et al. 2012) was used to run Bayesian analyses. In the process, four Markov Chain Monte Carlo (MCMC) chains were used to run 10,000,000 generations at the same time with a sampling frequency of 1000 generations and dividing 25% of the trees as burn-in. Branch support was evaluated through standard statistical testing (bootstrap support and posterior probability).

**Table 2.** Vouchers of *Mecistocephalus* species and outgroup and their GenBank accession numbers.

No.	Species	Voucher	Locality	COI	Reference
1	*M.chuensis* sp. nov.	CMMI 20200121008	Hengyang, Hunan, China	OR864658	This study
2	*M.chuensis* sp. nov.	CMMI 20210408124	Wuhan, Hubei, China	OR864659	This study
3	*M.chuensis* sp. nov.	CMMI 20210409130	Jinmen, Hubei, China	OR864660	This study
4	*M.diversisternus*	−	−	AB610776.1	Chao and Chang (unpublished)
5	*M.guildingii*	−	−	AF370837.1	Giribet et al. 2001
6	*M.guildingii*	CMMI 20201214103	Nantong, Jiangsu, China	OR864662	This study
7	*M.guildingii*	CMMI 20200608031	Yuanjiang, Hunan, China	OR864663	This study
8	*M.huangi* sp. nov.	CMMI 20230705004D	Gejiu, Yunnan, China	OR864653	This study
9	*M.huangi* sp. nov.	CMMI 20230830001D	Gejiu, Yunnan, China	OR864654	This study
10	*M.huangi* sp. nov.	CMMI 20201022122	Honghe Hani and Yi Autonomous Prefecture, Yunnan, China	OR864655	This study
11	*M.japonicus*	−	−	AB610480.1	Chao and Chang (unpublished)
12	*M.marmoratus*	−	−	AB672644.1	Chao and Chang (unpublished)
13	*M.marmoratus*	−	−	AB672611.1	Chao and Chang (unpublished)
14	*M.marmoratus*	−	−	AB610773.1	Chao and Chang (unpublished)
15	*M.marmoratus*	−	−	AB610772.1	Chao and Chang (unpublished)
16	*M.marmoratus*	−	−	AB610501.1	Chao and Chang (unpublished)
17	*M.marmoratus*	−	−	AB610500.1	Chao and Chang (unpublished)
18	*M.multidentatus*	−	−	AB610774.1	Chao and Chang (unpublished)
19	*M.multidentatus*	−	−	AB672610.1	Chao and Chang (unpublished)
20	*M.multidentatus*	−	−	AB610775.1	Chao and Chang (unpublished)
21	*M.multidentatus*	CMMI 20201205104	Shenzhen, Guangdong, China	OR864661	This study
22	*M.multidentatus*	CMMI 20230307001D	Shenzhen, Guangdong, China	OR864664	This study
23	*M.mikado*	CMMI 20200515001	Laibin, Guangxi Zhuang Autonomous Region, China	OR864668	This study
24	*M.mikado*	CMMI 20190418026	Laibin, Guangxi Zhuang Autonomous Region, China	OR864669	This study
25	*M.rubriceps*	CMMI 20210419105	Chengdu, Sichuan, China	OR864666	This study
26	*M.smithii*	CMMI 20201108126	Nanjing, Jiangsu, China	OR864667	This study
27	*M.smithii*	CMMI 20191031042	Zhoushan, Zhejiang, China	PP101253	This study
28	*Tygarrupjavanicus*	−	−	KM491598.1	Thormann and von der Mark (unpublished)

## ﻿Results

### ﻿Molecular phylogenetic analyses and species delimitation

Both the PTP and ABGD methods classified *M.huangi* sp. nov. and *M.chuensis* sp. nov. as distinct species. Eleven candidate species were identified using the ABGD method (0.001 < P < 0.037927). Using the PTP (ML) and PTP (BI) methods, 17 and 18 units were identified, respectively. Furthermore, Kimura two-parameter (K2P) distances between *Mecistocephalus* species ranged from 15.6% (*M.smithii* against *M.diversisternus*) to 23.9% (*M.chuensis* sp. nov. against *M.guildingii* Newport, 1843), and the average K2P genetic distance was 19.8% (Table 3). The intraspecific divergence within *Mecistocephalus* taxa was low, ranging from 0.5% (*M.multidentatus* Takakuwa, 1936) to 5.7% (*M.mikado* Attems, 1928). The mean distance within *M.chuensis* sp. nov. was second highest at 5.0% (Table 4). The K2P genetic distance between the two new species was 18.4%, well above the upper limit for intraspecific genetic distance. Considering the morphological characteristics of the samples and intraspecific genetic distances, both PTP methods (ML and PI) likely overestimated the number of species within *Mecistocephalus*.

**Table 3.** Mean K2P genetic distance between the *Mecistocephalus* species based on COI sequences.

		(1)	(2)	(3)	(4)	(5)	(6)	(7)	(8)	(9)
**(1)**	*M.multidentatus*									
**(2)**	*M.guildingii*	21.0%								
**(3)**	*M.marmoratus*	18.8%	21.5%							
**(4)**	*M.diversisternus*	18.8%	20.9%	19.1%						
**(5)**	*M.japonicus*	18.2%	20.8%	17.6%	16.9%					
**(6)**	*M.huangi* sp. nov.	17.5%	20.9%	18.6%	20.3%	20.1%				
**(7)**	*M.smithii*	18.0%	18.3%	17.3%	15.6%	16.4%	18.1%			
**(8)**	*M.chuensis* sp. nov.	18.1%	23.9%	20.6%	19.2%	19.7%	18.4%	21.3%		
**(9)**	*M.rubriceps*	21.0%	22.7%	19.0%	23.0%	19.1%	21.1%	18.6%	23.6%	
**(10)**	*M.mikado*	21.0%	23.5%	21.3%	22.2%	17.2%	19.3%	19.2%	23.4%	21.3%

**Table 4.** Mean K2P genetic distance within the *Mecistocephalus* species based on COI sequences.

Examined species	Mean distance	Standard error
*M.multidentatus*	0.5%	0.2%
*M.guildingii*	0.8%	0.3%
*M.marmoratus*	3.6%	0.7%
*M.diversisternus*	-	-
*M.japonicus*	-	-
*M.huangi* sp. nov.	2.0%	0.5%
*M.smithii*	1.6%	0.6%
*M.chuensis* sp. nov.	5.0%	0.8%
*M.rubriceps*	-	-
*M.mikado*	5.7%	1.0%

**Figure 2.**
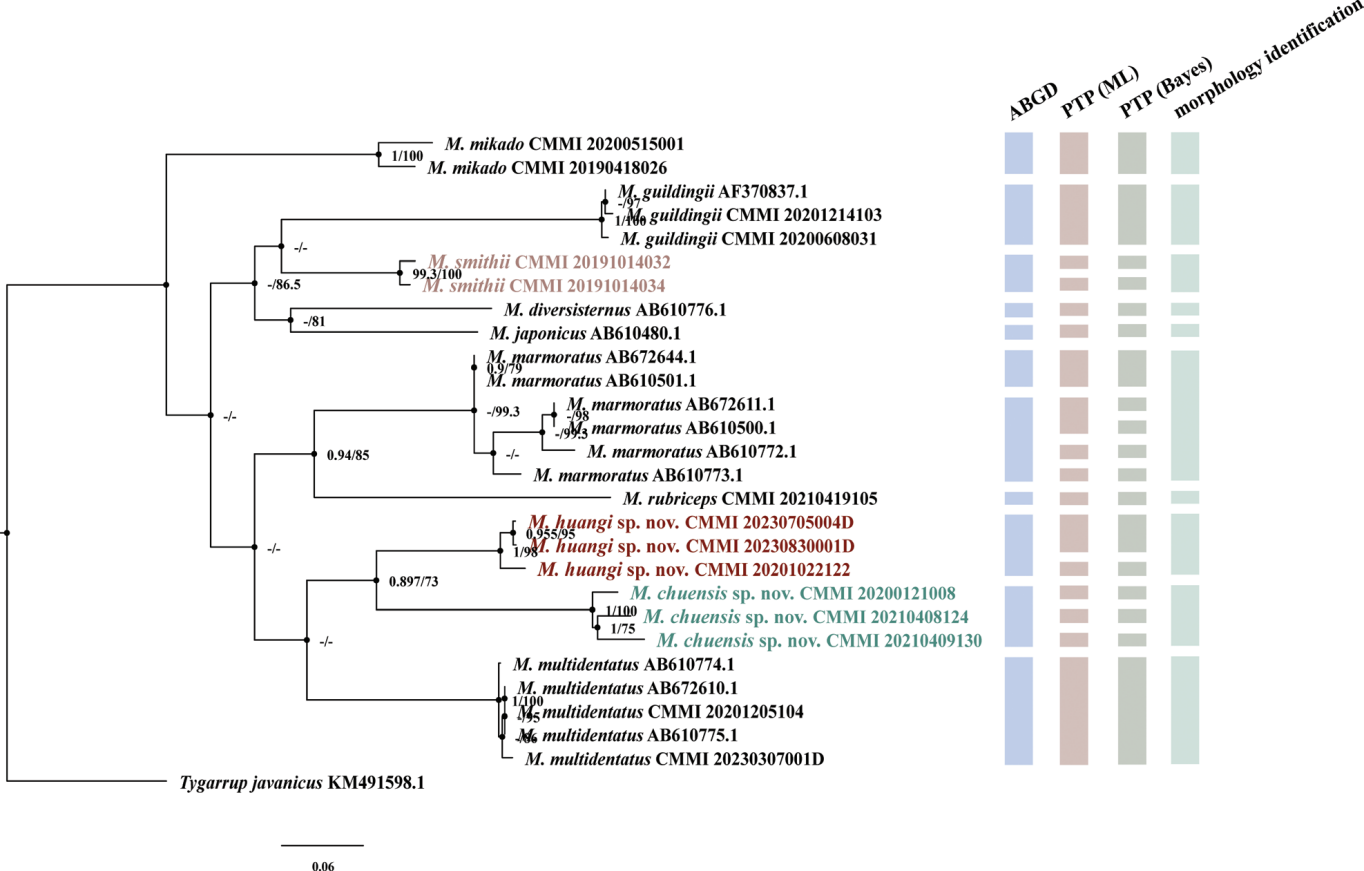
Phylogenetic tree based on COI for *Mecistocephalus* with Bayesian posterior probability (PP > 0.9 / BS > 70%, PP marked in the left and BS in the right) values and bootstrap support following ML analysis for each node and the result of species delimitation of single locus COI are based on using three species delimitation methods ABGD, PTP(ML), and PTP(BI).

A phylogenetic analysis and the ABGD method revealed 11 species. For *M.marmoratus*, the observed internal branch length was consistent with multiple taxonomic units identified though ABGD and PTP delimitation but disagreed with results based on morphological characters (Fig. 2). The two new species described here were recovered as sister taxa, with moderate support in phylogenetic analyses (Posterior Probability, PP = 0.897; Bootstrap Support, BS = 73%). In the phylogeny, a basal dichotomy was observed between *M.mikado* and the remaining species. All other *Mecistocephalus* taxa were resolved into three clades. *Mecistocephalushuangi* sp. nov. and *M.chuensis* sp. nov. were recovered as a clade with *M.multidentatus*, sister to a clade containing *M.marmoratus* and *M.rubriceps*. The remaining species, *M.guildingii*, *M.smithii*, *M.diversisternus*, and *M.japonicus*, all formed one clade sister to the other two. Internal structure was observed. Notably, samples from Hubei Province (CMMI 20210408124, 20210409130) within *M.chuensis* sp. nov. were separated from those from Hunan Province (PP = 1, BS = 100%). Similarly, samples from Gejiu City (CMMI 20201022122) and Jianshui County (CMMI 20230705004D, 20230830001D) in Yunnan Province, identified as *M.huangi* sp. nov., exhibited clear differentiation into two branches, with high nodal support (PP = 1, BS = 98%). Conversely, samples collected from the same geographic site exhibited relatively small intraspecific distances, as exemplified by *M.smithii* and *M.chuensis* sp. nov. in PTP analyses.

### ﻿Taxonomic accounts

**Family Mecistocephalidae Bollmann, 1893**

#### ﻿Genus Mecistocephalus

Taxon classificationAnimaliaGeophilomorphaMecistocephalidae

Newport, 1843

88047F60-283E-520F-BDE5-7B15E4FF76D0

##### Diagnosis.

Mecistocephalids with at least 45 leg-bearing segments. Two clypeal plagulae, separated by a mid-longitudinal stripe. Spiculum usually present. Buccae with setae at least in the posterior half. Posterior alae of the labrum smooth. Coxosternum of the first maxillae divided, with a mid-longitudinal suture. Coxosternum of the second maxillae undivided; groove from the metameric pore reaching the lateral margin of the coxosternum. Telopodites of the second maxillae well developed, overreaching those of the first maxillae; pretarsus present. Forcipular trochanteroprefemur with a distal tooth and often with another tooth at approximately mid-length. Sternal sulcus of trunk segments furcate or not. Last leg-bearing segment: Coxopleura usually without a macropore distinct from other pores; legs usually as slender in males as in females, with or without one or two short apical spines (Uliana et al. 2007).

#### ﻿ Mecistocephalus chuensis

Taxon classificationAnimaliaGeophilomorphaMecistocephalidae

Jiang & You sp. nov.

123FE343-C5CC-500D-BB00-E5FECECCD806

https://zoobank.org/CBECBDD2-1928-49AB-ADAA-859E4D7D74AA

Figs 3 , 4

##### Material examined.

***Holotype*.** • ♂; (CMMI 20210409134); **China, Hubei Province**, Jingshan County, Kongshandong Scenic Area; 30.9735°N, 113.0377°E; 110 m a.s.l.; 9 Apr. 2021; coll. Tianyun Chen & Zhidong Wang. ***Paratypes*.** • 2 ♂♂, 2 ♀♀; (CMMI 20210409130−133); same data as holotype. • 1 ♂, 7 ♀♀; (CMMI 20210408121−20210408124, -129, -131, -132, -134); **Hubei Province, Wuhan**, Ma’anshan Forest Park; 30.5146°N, 114.4394°E; 110 m a.s.l.; 8 Apr. 2021; coll. Tianyun Chen & Zhidong Wang. • 1 ♀ (CMMI 202100121008); **Hunan Province**, **Hengyang**, Chuanshan Ave; 30.5146°N, 114.4394°E; 120 m a.s.l.; 12 Jan. 2021; coll. Chao Jiang.

##### Diagnosis.

A *Mecistocephalus* species with 49 leg pairs. Head length-to-width ratio 1.77, each side of clypeus with five or six smooth insulae, clypeal ratio (areolate part/ non-areolate part) of 1.22, sensilla on plagulae absent, posterior 1/2 of cephalic pleurite bearing a group of setae, forcipular cerrus composed of two paramedian rows of setae, mandible with ~ 8 well-developed lamellae, and first lamella with seven teeth. Sternal sulcus furcated at an obtuse angle.

##### Description.

***Holotype*** (CMMI 20210409134).

Body length: 58 mm; posterior part slightly slender. Head and forcipular segment dark red in color; remainder yellow.

**Figure 3.**
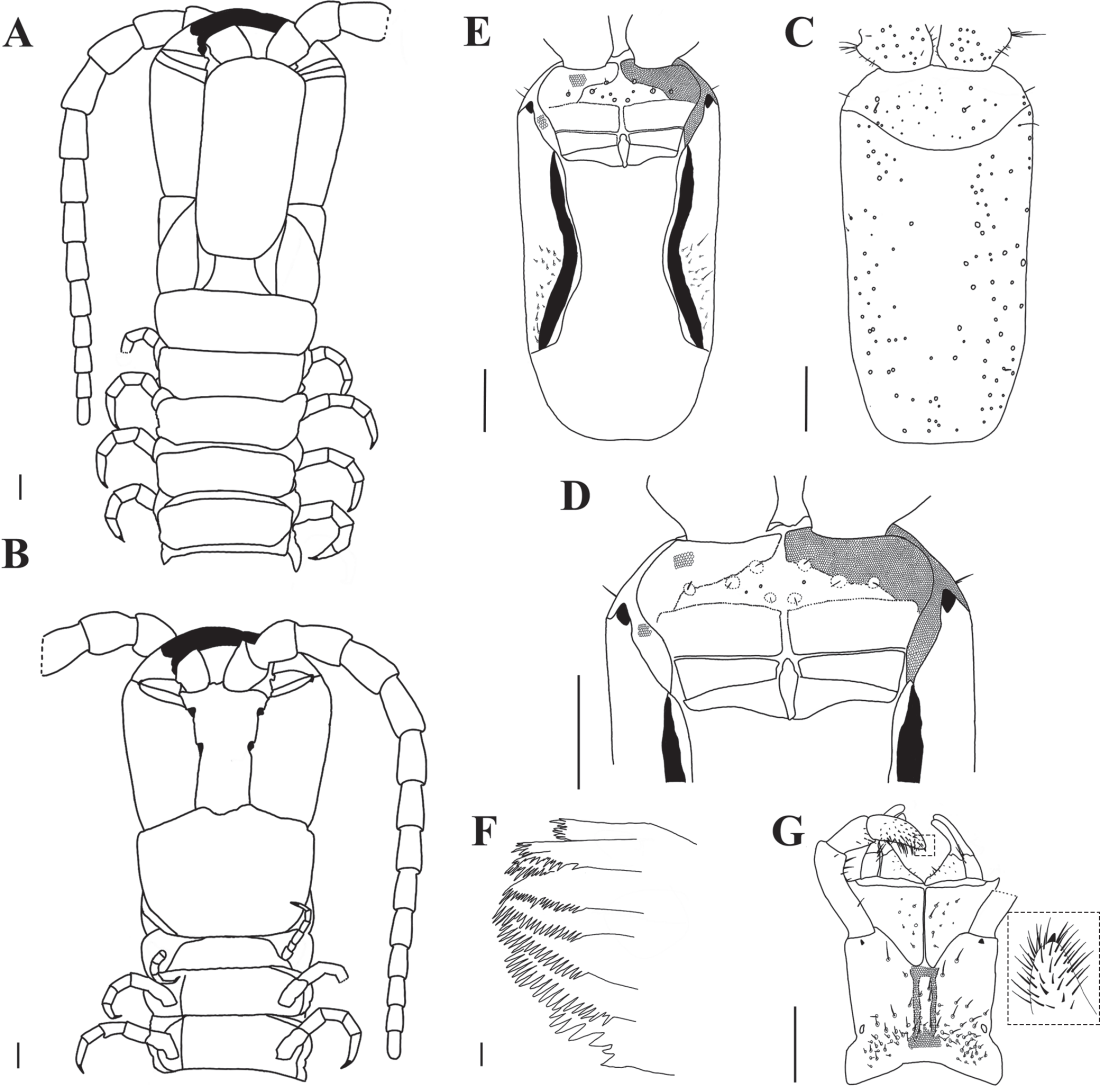
*Mecistocephaluschuensis* Jiang & You, sp. nov., holotype (CMMI 20210409134) **A** anterior part of body, dorsal view (right antenna not drawn) **B** anterior part of body, ventral view (right antenna not drawn) **C** cephalic plate, dorsal view **D** anterior part of head, ventral view (maxillae removed) **E** cephalic plate, ventral view (maxillae removed) **F** mandible **G** maxillae and inner illustration shows the telopodite article III of the second maxillae, ventral view (right telopodite of the second maxillae removed) Part of areolation not drawn. Scale bars: 20 μm (**F**); 1 mm (**A−E, G**).

***Cephalic plate*** (Fig. 3A−C): sub-rectangular, length-to-width ratio of 1.77; lateral margins slightly convergent backward, strongly convergent backward at proximal three-fourths, maximum width 2.88 mm; transverse suture protrudes to the back edge of the cephalic plate in an arc; two or three setae in the anterior of back side, punctate depressions interspersed throughout. Antennae 5.3 × as long as the head width. Apical sensilla ~ 8 μm long.

***Clypeus*** (Fig. 3D): clypeal ratio (areolate part/ non-areolate part) ~ 1.22; five or six insulae on each side of clypeus, only four bearing setae on each side; the transverse suture of clypeal plagulae almost straight, sensilla inside plagulae absent.

***Labrum*** (Fig. 3D): anterior ala with medial margin not reduced to a vertex, medial margin ~ 1/3 of the length of posterior ala; mid piece protruding forward over side pieces; posterior margin of side pieces sinuous, concave or convex with respect to straight anterior margin; the hair-like fringes and projections on the labral side pieces absent, the comma-shaped sclerite lateral to the labral side pieces present.

***Cephalic pleurite*** (Fig. 3E): spiculum present, group of setae only on the posterior 1/2.

***Mandible*** (Fig. 3F): approximately eight well-developed lamellae; first lamella with seven teeth; average intermediate lamella with ~ 22 teeth; basal teeth as same size as distal teeth; basal tooth large, shorter than the teeth of the first lamella.

***First maxillae*** (Fig. 3G): antero-external corners of coxosternite protruding and short; coxosternite divided by mid-longitudinal sulcus, with eight or nine setigerous insulae on each side; coxal projection 1.2 × wider than long, eleven setae on medial marginal and clavate lappet present; telopodites 2.86 × longer than wide, clavate lappet present.

***Second maxillae*** (Fig. 3G): sclerotic ridge on the middle of coxosternite circumscribing three or four setigerous insulae; each side of coxosternite with setae on the lateral to each metameric pore; telopodite article I 1.4 × longer than wide; anterior end of article II with six surrounding setae; article III 2.78 × longer than wide, with distal end densely setose, pretarsus present, conical in shape.

**Figure 4.**
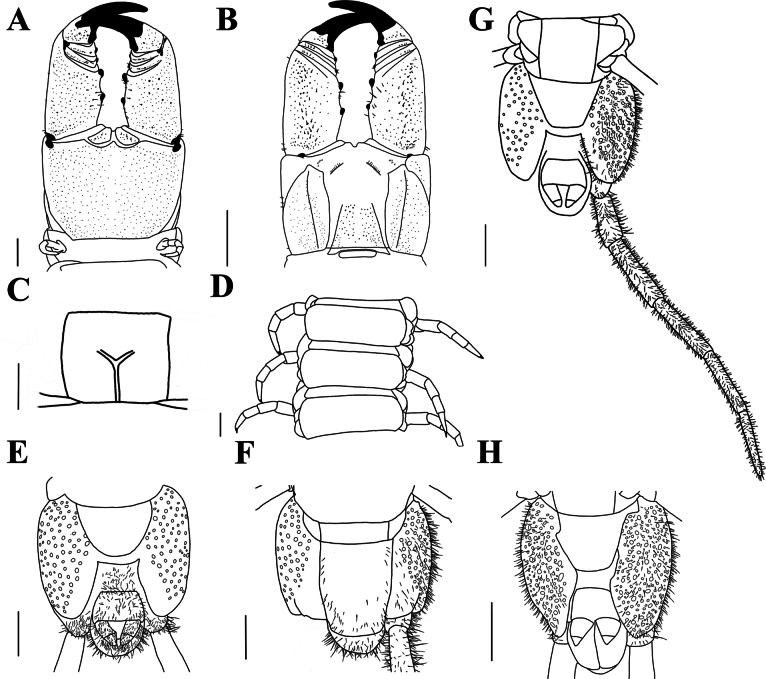
*Mecistocephaluschuensis* Jiang & You, sp. nov., holotype (CMMI 20210409134) **A** forcipular segment, ventral view **B** forcipular segment, dorsal view **C** sternite of leg-bearing segment VI, ventral view **D** tergites of leg-bearing segments VI−VIII, dorsal view **E** ultimate leg-bearing segment, ventral view **F** ultimate leg-bearing segment, dorsal view **G** ultimate leg-bearing segment and left leg, ventral view **H** ultimate leg-bearing segment, ventral view (paratype CMMI 20210408122). Scale bars: 500 μm (**H**); 1 mm (**A−G**).

***Forcipular segment*** (Fig. 4A, B): exposed part of the coxosternite with a width-to-length ratio of 0.68; cerrus composed of two convergent rows of setae and a pair of setae on each side. Forcipular trochanteroprefemur length-to-width ratio of 1.7, proximal tooth slightly smaller than the distal tooth; femur and tibia each with one tooth, equal in size; tarsungulum with two dark brown, small. basal teeth, one dorsal to the other.

***Leg-bearing segments*** (Fig. 4C, D): 49 leg-bearing segments; a few sternites with sternal sulcus and dispersed setae; sternal sulcus of anterior segments furcate, branches at an obtuse angle.

***Ultimate leg-bearing segment*** (Fig. 4E–H): metasternite trapezoidal, length-to-width ratio of 1; each coxopleuron covered with dense pore-field into the distal end ultimate leg telopodite with short setae, apical claw absent.

***Postpedal segments*** (Fig. 4E, G, H): male gonopods tapered and biarticulated.

##### Variation in paratypes.

Body length up to 64 mm, cephalic plate length-to-width ratio of 1.77–1.97, antennae length-to-head width ratio of 4.8–5.5, medial projection of first maxillae width-to-length ratio of 1.2–2.14 and telopodites length-to-width ratio of 1.43–2.86, telopodites article I of second maxillae length-to-width ratio of 4–6.88, article III ratio of 2.78–3.18, forcipular trochanteroprefemur length-to-width ratio of 1.27–1.7, exposed part of coxosternite length-to-width ratio of 0.68–0.79. Female gonopods also biarticulated.

##### Remarks.

As shown in Table 5, *M.megittii* Verhoeff, 1937, *M.stenoceps* Chamberlin, 1944, *M.enigmus* Chamberlin, 1944 and *M.chuensis* sp. nov. are similar in the numbers of leg-bearing segments, furcate sternal sulcus and clypeal ratio, but other features distinguish them. *Mecistocephalusmegittii* notably differs from *M.chuensis* sp. nov. in the presence of a large tooth on the tarsungulum (only a small tooth at the base of the tarsungulum in the latter) and the presence of one or two setae on each side of clypeus (3 setae on each side of clypeus in the latter) (Attems 1947).

**Table 5.** Distinguishing characteristics of new species from similar species, incorporating data from Attems (1947), Chamberlin (1944), Matic and Dărăbanțu (1969), original descriptions, and re-descriptions.

	*M.chuensis* sp. nov.	*M.huangi* sp. nov.	*M.megittii* Verhoeff, 1937	*M.stenoceps* Chamberlin, 1944	*M.enigmus* Chamberlin, 1944	*M.lanzai* Matic & Dărăbanțu, 1969
Locality	Hunan, China	Yunnan, China	Rangoon, Myanmar	Purmerend, Batavia Bay	Poentjak, Java	Giohar
Number of leg-bearing segments	49	49	49	49	49	49
Head length-to-width ratio	1.77	2	2.5	2.03	1.73	1.58
Clypeal ratio	1.22	1	1.1	1.47		1.63
Setae on each side of clypeus	4	2–3	1–2	4		3
Cephalic pleurite	only on the posterior 1/2	only on the posterior 1/2				only on the posterior 1/2
Mandible with well-developed lamellae	8	6			~ 9 or 10	9 or 10
Forcipules	trochanteroprefemur with both basal and distal teeth	trochanteroprefemur with both basal and distal teeth	tooth on tarsungulum very prominent	trochanteroprefemur with both basal and distal teeth	femur without tooth	trochanteroprefemur with both basal and distal teeth
Sternal sulcus	furcate	furcate	furcate		furcate	furcate
Metasternite of ultimate leg-bearing segment	trapezoid	with a pillow-like protrusion		significantly narrow, the posterior end rather narrowly rounded		

Similarly, the other two species and *M.chuensis* sp. nov. can be distinguished by the location of the setae and presence or absence of a tooth on the forcipular femur. The clypeus of *M.stenoceps* Chamberlin has a series of three setae in a transverse row on each side farther anteriorly than in *M.chuensis* sp. nov., and *M.enigmus* lacks a femoral tooth (Chamberlin 1944), which is present in *M.chuensis* sp. nov.

##### Distribution.

China (Hubei, Hunan).

##### Etymology.

The specific name is derived from its distribution in Hunan Province and Hubei Province, where “Chu” in ancient China usually referred to these regions.

#### ﻿ Mecistocephalus huangi

Taxon classificationAnimaliaGeophilomorphaMecistocephalidae

Jiang & You sp. nov.

31CC1BCC-C407-5744-810F-3E963D4E4FFF

https://zoobank.org/5654176E-38FF-4C11-9F9C-9B20CEE6DDA7

Figs 5 , 6

##### Material examined.

***Holotype*.** • ♂; (CMMI 20201022122); **China, Yunnan Province**, Honghe Hani and Yi Autonomous Prefecture, Jianshui County, Yanzidong Scenic Area; 23.6359°N, 103.0537°E; 1260 m a.s.l.; 22 Oct. 2021; coll. Chao Jiang & Zhidong Wang. ***Paratypes*.** • 2 ♂♂, 4 ♀♀; (CMMI 20201022119, -121, -123, -125, -126, -137); same data as holotype; 22 Oct, 2021. • 3 ♂♂, 4 ♀♀; (CMMI 20190122001D−007D); **Yunnan Province**, **Gejiu**, Baohua Park; 23.3545°N, 103.1621°E; 1770 m a.s.l.; 22 Jan. 2019; coll. Huiqin Ma; • 2 ♂♂; (CMMI 20230705004D, 20230705005D); same location but collected at 5 Jul. 2023; coll. Yangyang Pan & Jiabo Fan. • 1 ♂, 8 ♀♀; (CMMI 20230830001D−20230830009D); same location but collected at 30 Aug. 2023; coll. Tianyun Chen & Jiabo Fan.

##### Diagnosis.

A *Mecistocephalus* species with 49 leg pairs. Head length-to-width ratio 2, each side of clypeus with two or three smooth insulae, clypeal ratio (areolate part/ non-areolate part) ~ 1, plagulae without sensilla, posterior 1/2 of cephalic pleurite bearing a group of setae, mandible with ~ 6 well-developed lamellae and first lamella with ~ 6 teeth. Sternal sulcus furcated at an obtuse angle. Metasternite trapezoid and with a pillow-like protrusion.

##### Holotype description.

Body length 69 mm; the posterior part slightly slender; head and forcipular segment dark brown in color; remainder yellow.

**Figure 5.**
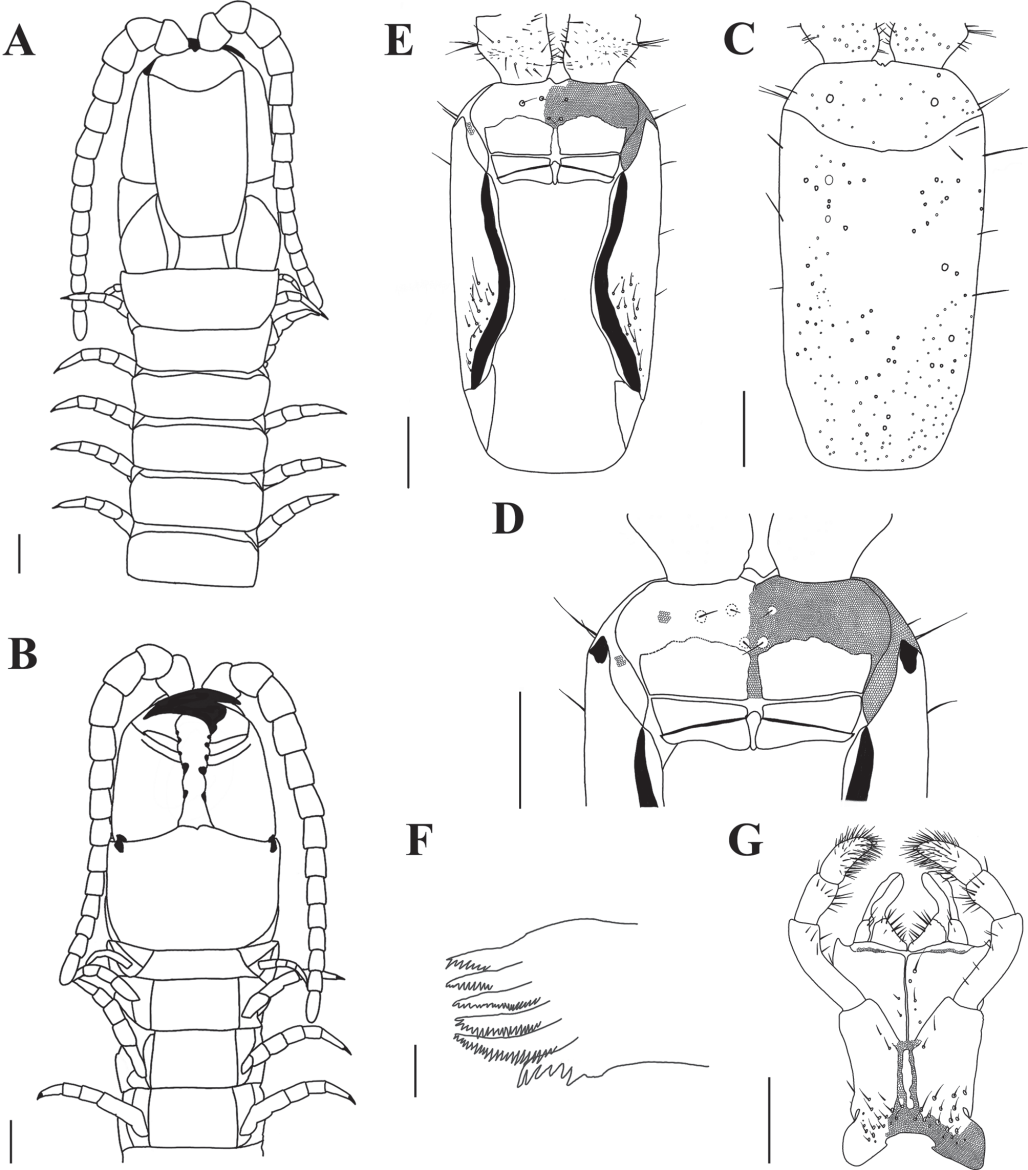
*Mecistocephalushuangi* Jiang & You, sp. nov., holotype (CMMI 20201022122) **A** anterior part of body, dorsal view **B** anterior part of body, ventral view **C** cephalic plate, dorsal view **D** anterior part of head, ventral view (maxillae removed) **E** cephalic plate, ventral view (maxillae removed) **F** mandible **G** maxillae, ventral view. Scale bars: 50 μm (**F**); 1 mm (**A–E, G**). Part of areolation not drawn.

***Cephalic plate*** (Fig. 5A–C): sub-rectangular, length-to-width ratio 2; lateral margins slightly convergent backward, strongly convergent backward at proximal four-fifths, the maximum width 2.58 mm; transverse suture protruding to the back edge of the cephalic plate in an arc; six or seven setae on each side of back, punctate depressions interspersed throughout; dorsal side of cephalic plate with scattered puncta. Antennae 5.35 × as long as the head width. Apical sensilla at the actual distal apex of article XIV ~ 8 μm long.

***Clypeus*** (Fig. 5D): clypeal ratio (areolate part/ non-areolate part) ~ 1; each side bearing two or three insulae, each circumscribing one seta; transverse suture of the clypeal plagulae slightly protruding from the front cephalic plate, concave inward near cephalic pleurite, sensilla absent.

***Labrum*** (Fig. 5D): anterior ala medial margin ~ 5/12 of length of posterior ala; the middle piece protrudes forward into a vertex over side pieces; posterior margin of side pieces curved, concave with respect to straight anterior margin; the hair-like fringes and projections on the labral side pieces absent, the comma-shaped sclerite lateral to the labral side pieces present.

***Cephalic pleurite*** (Fig. 5E): spiculum present; a group of setae only on the posterior 1/2.

***Mandible*** (Fig. 5F): approximately six well developed lamellae; first lamella with six teeth; average intermediate lamella with ~ 22 teeth, all teeth of similar size, the region before the first lamella without teeth.

***First maxillae*** (Fig. 5G): antero-external corners of coxosternite protruding and short; coxosternite divided by mid-longitudinal sulcus, three to four setigerous insulae on each side; coxal projections 1.1 × as wide as long, nine setae on medial margin; clavate lappets present on coxal projections; telopodites 2.14 × as long as wide, clavate lappet present.

***Second maxillae*** (Fig. 5G): sclerotic ridge on the middle of coxosternite with two setigerous insulae on the posterior ridge; each side of coxosternite with at least 17 setigerous insulae on the posterior side; telopodite article I 3.85 × as long as wide, the ventral and dorsal part of anterior telopodites both with five vertical setae; anterior article II with ten surrounding setae; article III 2.75 × as long as wide, apex densely setose, pretarsus present.

**Figure 6.**
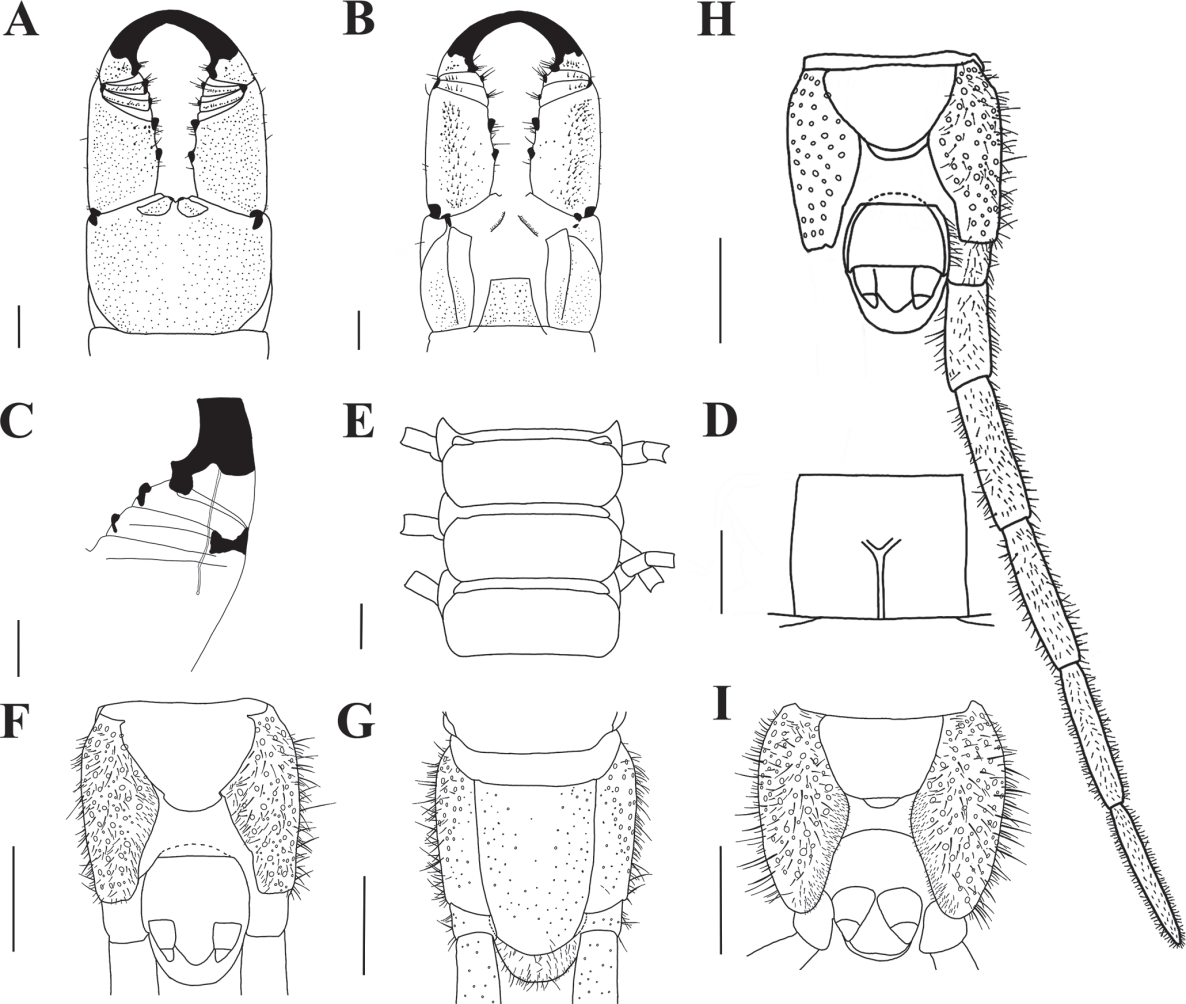
*Mecistocephalushuangi* Jiang & You, sp. nov., holotype (CMMI 20201022122) **A** forcipular segment, ventral view **B** forcipular segment, dorsal view **C** part of right forcipule, ventral view **D** sternite of leg-bearing segment VI, ventral view **E** tergites of leg-bearing segments VI–VIII, dorsal view **F** ultimate leg-bearing segment, ventral view **G** ultimate leg-bearing segment, dorsal view **H** ultimate leg-bearing segment and left leg, ventral view **I** ultimate leg-bearing segment, ventral view (paratype CMMI 20230803003D). Scale bars:100 μm (**C**); 500 μm (**I**); 1 mm (**A, B, D−H**).

***Forcipular segment*** (Fig. 6A–C): exposed part of coxosternite width-to-length radio 1.4; cerrus only composed of two convergent rows of setae. Trochanteroprefemur length-to-width radio 1.43, proximal tooth slightly smaller than the distal tooth; both femur and tibia with one tooth and the former smaller than the latter; tarsungulum with two dark brown and small basal teeth, one dorsal to the other; poison calyx reaching the distal part of trochanteroprefemur.

***Leg-bearing segments*** (Fig. 6D, E): 49 leg-bearing segments; a few sternites in the anterior part of leg-bearing segment with sternal sulcus and dispersed setae; sternal sulcus of anterior segments furcate, branches at an obtuse angle; the first pair of legs much smaller than the rest, apical claw simple.

***Ultimate leg-bearing segment*** (Fig. 6F–I): metasternite trapezoid in shape and with a small pillow-like protrusion in the end, length-to-width radio of 1.45; each coxopleura covered by a dense pore-field except for the distal end; ultimate leg telopodite with short setae, apical claw absent; the last leg-bearing segment covered with setae.

***Postpedal segments*** (Fig. 6F, H, I): male gonopods tapered and biarticulated.

##### Variation in paratypes.

Body length up to 69 mm, cephalic plate length-to-width ratio 1.84–1.97, antennae length to head width ratio 3.57–5.17, medial projection of first maxillae width to length ratio 1.07–1.29 and telopodites length to width ratio 2.5–4.8, telopodites article I of second maxillae length-to-width radio 3.43–4.29, article III ratio 4–4.29, forcipular trochanteroprefemur length-to-width ratio 1.16–1.19, exposed part of coxosternite length to width ratio 0.69–0.75. The end of female metasternite also with a small pillow-like protrusion. Females gonopods also biarticulated.

##### Remarks.

This new species resembles *M.lanzai* Matic & Dărăbanțu (1969) in the furcate sternal sulci, three pairs of setae on the clypeus, and a cluster of setae located exclusively on the posterior 1/2 of the cephalic pleurite (Table 5). However, a distinguishing feature of *M.lanzai* is the absence of a basal tooth on the tarsungulum.

*Mecistocephalushuangi* sp. nov. exhibits a clear morphological resemblance with *M.chuensis* sp. nov. However, it can be differentiated from the latter by the presence of five to six smooth insulae on the clypeus of the former and a pillow-like protrusion on the metasternite which is common in both paratypes and non-type material of *M.huangi* sp. nov. (Figs 5E, 6F–I). Furthermore, this species and *M.chuensis* sp. nov. were found to form two distinct but closely related clades in the phylogenetic analysis (Fig. 2).

##### Distribution.

China (Yunnan).

##### Etymology.

The specific name is dedicated to Dr. Luqi Huang for his generous help in the intensive fieldwork for collecting specimens of geophilomorphs.

#### ﻿ Mecistocephalus smithii

Taxon classificationAnimaliaGeophilomorphaMecistocephalidae

Pocock, 1895

95DA3223-887B-54F9-AE1B-38313CBAE772

Figs 7 , 8

Mecistocephalus smithii Pocock, 1895: 351.

##### Material examined.

• 2 ♂♂, 5 ♀♀; (CMMI 20191014012, −032, 20191014034−20191014036); **China, Guangdong Province**, Guangzhou, Maofengshan Forest Park; 23.2983°N, 113.4644°E; 330 m a.s.l.; 14 Oct. 2019; coll. Chao Jiang. • 2 ♀♀; (CMMI 20201217111, 20201217112); **Guangdong Province, Heyuan**, Xinfengjiang Reservoir; 23.7703°N, 114.6300°E; 180 m a.s.l.; 17 Dec. 2020; coll. Zhidong Wang. • 1 ♀; (CMMI 20191031042); **Zhejiang Province, Zhoushan**, Changgangshan Forest Park; 30.0355°N, 122.1201°E; 130 m a.s.l.; 31 Oct. 2019; coll. Chao Jiang. • 2 ♂♂, 1 ♀; (CMMI 20201108123, -124, -126); **Jiangsu Province, Nanjing**, Fuguishan Forest Park; 32.1006°N, 118.586°E; 130 m a.s.l.; 8 Nov. 2020; coll. Zhidong Wang.

##### Diagnosis.

A *Mecistocephalus* species with 59 leg pairs. Head length-to-width ratio ~ 1.7, each side of clypeus with ~ 20–22 setae, clypeal ratio (areolate part/ non-areolate part) of ~ 1, plagulae with sensilla, cephalic pleurite without setae, forcipular coxosternite only with a part of short cerrus. Sternal sulcus with short branches.

##### Re-description.

Body length 74–88 mm (limited to the above samples); posterior part slightly slender. Head and forcipular segment dark red in color; remainder yellow.

**Figure 7.**
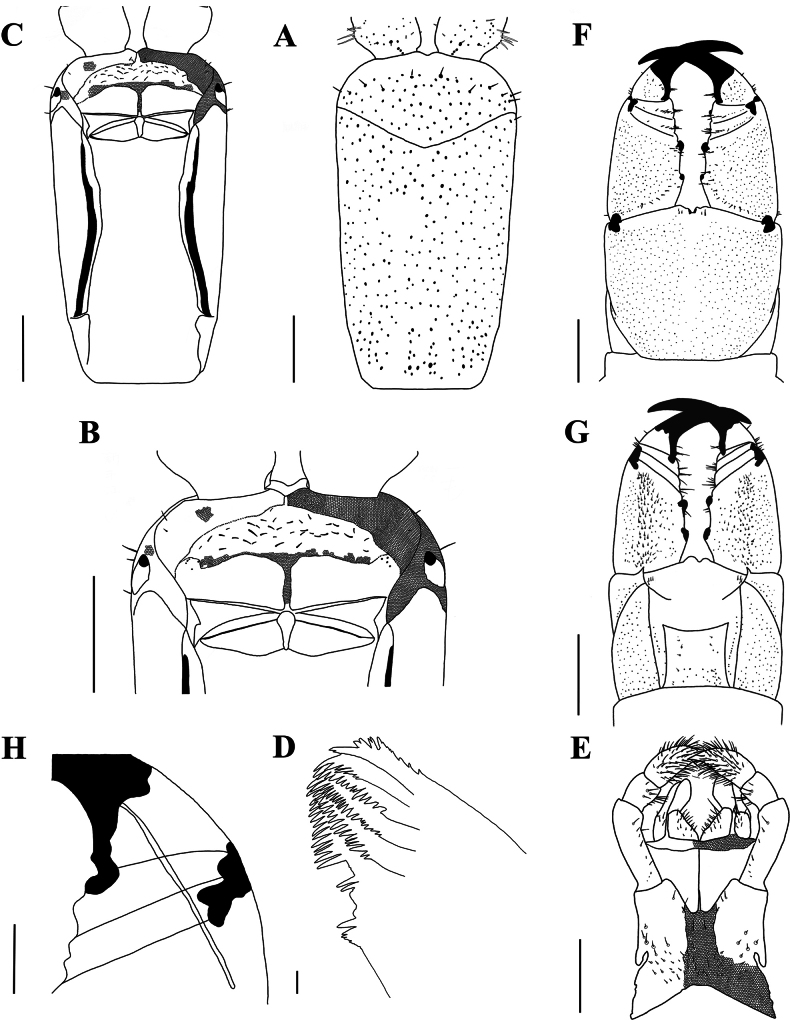
*Mecistocephalussmithii* Pocock, 1895 (spm. CMMI 20191014031) **A** clypeus and cephalic pleurite, ventral view **B** anterior part of head, ventral view (maxillae removed) **C** cephalic plate, ventral view (maxillae removed) **D** mandible **E** maxillae, ventral view **F** forcipular segment, ventral view **G** forcipular segment, dorsal view **H** a part of right forcipule, ventral view. Part of areolation not drawn. Scale bars:20 μm (**D**), 100 μm (**H**); 1 mm (**A−C, E−G**).

***Cephalic plate*** (Fig. 7A): sub-rectangular, length-to-width ratio 1.7–2.1; lateral margins slightly convergent backward, the maximum width 2.65 mm; transverse suture protruded to the back edge of the cephalic plate, vertex pointed; suture line on the back side of the cephalic plate with four to six setae; dorsal cephalic plate with scattered puncta. Antennae 5.3 × as long as the head width. Apical sensilla ~ 10 μm long.

***Clypeus*** (Fig. 7B): clypeal ratio (areolate part/ non-areolate part) ~ 1; each side with 20–22 setae; the transverse suture of clypeal plagulae slightly protrude from the front cephalic plate; plagulae with groups of small sensilla localized to anterolateral corners.

***Labrum*** (Fig. 7B): anterior ala medial marginal ~ 1/3 of posterior ala; middle piece protrudes forward into a vertex over side pieces; posterior line of side pieces curve, convex with respect to straight anterior margin; the hair-like fringes and projections on the labral side pieces absent, the comma-shaped sclerite lateral to the labral side pieces present.

***Cephalic pleurite*** (Fig. 7C): spiculum present; cephalic pleurite without setae.

***Mandible*** (Fig. 7D): approximately seven to eleven well developed lamellae; first lamella with ~ 5 teeth; average intermediate lamella with ~ 22 teeth; basal teeth small and protruding.

***First maxillae*** (Fig. 7E): antero−external corners of coxosternite protruding and short; coxosternite divided by mid−longitudinal sulcus; coxal projection 1–1.2 × as wide as long, 17 setae on medial margin and clavate lappet present; telopodites 2.5–3.4 × as long as wide, clavate lappet present.

***Second maxillae*** (Fig. 7E): one sclerotic ridge on the middle of coxosternite with ~ 8 setigerous insulae; telopodite article I 3.33–4.5 × as long as wide, the ventral and dorsal part of interior telopodites both with four vertical setae; anterior article II with nine surrounding setae; article III 2.5–3 × as long as wide, distal end densely setose, pretarsus present.

***Forcipular segment*** (Fig. 7F–H): exposed part of coxosternite width-to-length radio 0.8–0.85; cerrus composed of a pair of setae on each side. Forcipular Trochanteroprefemur length-to-width radio of 1.13–1.35, two teeth present; both femur and tibia with one tooth and the former smaller than the latter; tarsungulum with one dark brown and small basal tooth; poison calyx reaching the distal part of trochanteroprefemur.

**Figure 8.**
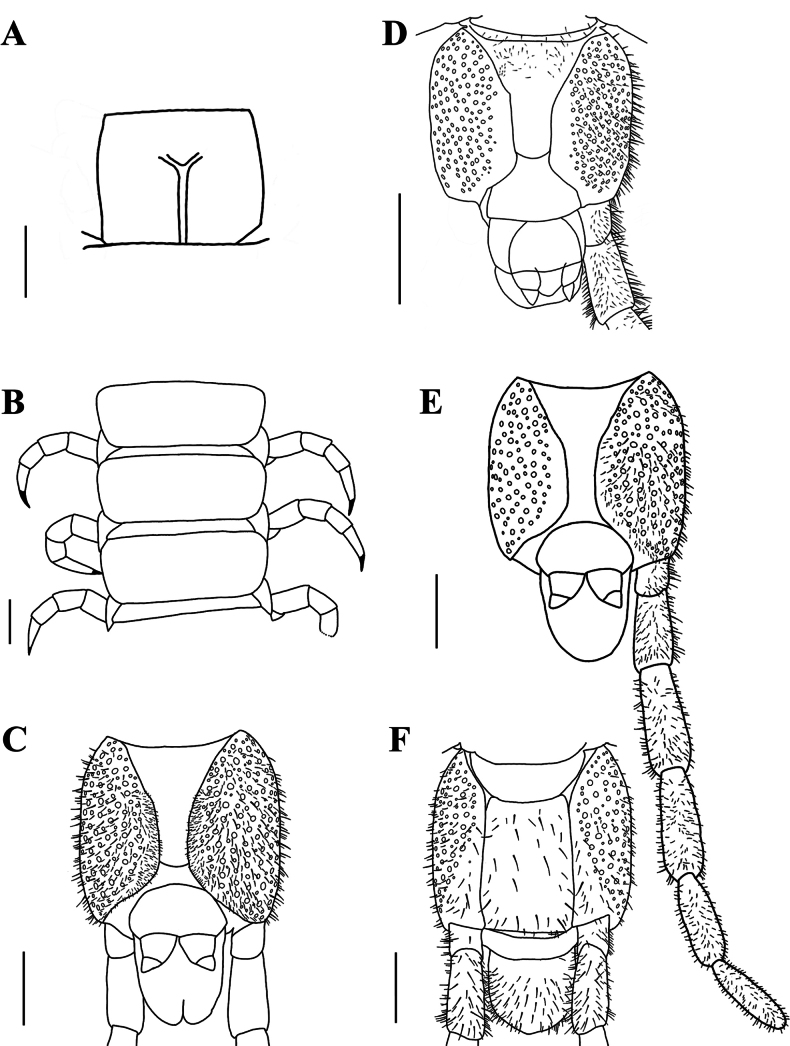
*Mecistocephalussmithii* Pocock, 1895 (spm. CMMI 20191014031) **A** sternite of leg-bearing segment VI, ventral view **B** tergite of leg-bearing segments VI–VIII, dorsal view **C** ultimate leg-bearing segment, ventral view **D** ultimate leg-bearing segment, ventral view (spm. CMMI 20191014012) **E** ultimate leg-bearing segment and left leg, ventral view **F** ultimate leg-bearing segment, dorsal view. Scale bars: 1 mm.

***Leg-bearing segments*** (Fig. 8A, B): 59 leg-bearing segments, a few sternite with sternal sulcus, almost all posterior sternite with setae; sternal sulcus of anterior segments furcate, and with short branches; the first pair of legs much smaller than the others, only one claw at the front.

***Ultimate leg-bearing segment*** (Fig. 8C, F): posterior line of metasternite protruding no more than intermediate sternite, sandwiched between two coxopleura; each of coxopleura covered with dense pore-fields except for the ends; ultimate leg-bearing with short setae, claw absent; setae distributed in various parts of the last leg-bearing segment.

***Postpedal segments*** (Fig. 8C, E, F): gonopods of both males and females tapered and biarticulated.

##### Remarks.

To date, within Asia, only two species of the genus *Mecistocephalus* possess 59 leg-bearing segments: *M.diversisternus* Silvestri, 1919 and *M.smithii* Pocock, 1895. It is noteworthy that some researchers have previously raised concerns regarding the accuracy of *M.smithii* records in Japan and Taiwan, suggesting that they may represent misidentification of *M.diversisternus* (Uliana et al. 2007). *Mecistocephalussmithii* was originally described from “Da-laen-Saen 30 miles S.W. of Ningpo” and “Wo Lee Lake, 25 miles S. of Ningpo” in the Chinese mainland. The locality “Da-laen-Saen 30 miles S.W. of Ningpo” is very likely “Dalei Shan” near (45 km SW) Ningbo, Zhejiang (Schawaller and Aston 2017), while the locality of “Wo Lee Lake, 25 miles S. of Ningpo” does not match any present geographical name in Ningbo after a survey among local residents. The original account was too brief, and the true identity of *M.smithii* remained unclear (Uliana et al. 2007).

To address this problem, we collected and examined new specimens near the type locality, as well as in other provinces. Therefore, we can confidently state that *M.smithii* is a distinct species to *M.diversisternus* and that it is present in China. The distinguishing features of *M.diversisternus* include a limited number of clypeal setae, typically less than ten, and a sternal sulcus that lacks bifurcation. In contrast, *M.smithii* has abundant setae on each side of the clypeus ranging from 20 to 22 and a bifurcate sternal sulcus with short branches.

##### Distribution.

China (Zhejiang, Guangdong).

### ﻿Key to the species of the *Mecistocephalus* in China

**Table:** 

1	Number of leg-bearing segments invariably 49	**2**
–	Number of leg-bearing segments not 49	**13**
2	Medial projection and telopodites of first maxillae longer than the telopodite of the second maxillae	***M.longichilatus* Takakuwa, 1936**
–	Medial projection and telopodites of first maxillae shorter than the telopodite of the second maxillae	**3**
3	Areolate part of the clypeus with smooth insulae	**4**
–	Areolate part of the clypeus without smooth insulae	**9**
4	Sternal sulcus furcate	**5**
–	Sternal sulcus not furcate	***M.rubriceps* Wood, 1862**
5	Posterior 1/2 of the cephalic pleurite bearing a group of setae	**6**
–	Both the anterior and posterior halves of the cephalic pleurite bearing a group of setae	***M.mikado* Attems, 1928**
6	Fewer than 6 smooth insulae on each side of clypeus	**7**
–	More than 6 smooth insulae on each side of clypeus	***M.multidentatus* Takakuwa, 1936**
7	More than 4 smooth insulae on each side of clypeus	***M.chuensis* sp. nov.**
–	Fewer than 4 smooth insulae on each side of clypeus	**8**
8	Each plagula covered with pore-like sensilla	***M.marmoratus* Verhoeff, 1934**
–	Plagulae without pore-like sensilla	***M.huangi* sp. nov.**
9	Clypeal ratio (areolate part/ non-areolate part) > 2	**10**
–	Clypeal ratio (areolate part/ non-areolate part) < 2	**12**
10	Sternal sulcus furcate	**11**
–	Sternal sulcus not furcate	***M.changi* Uliana, Bonato, Minelli, 2007**
11	Metasternite of ultimate leg-bearing segment approx. as wide as long	***M.ongi* Takakuwa, 1934**
–	Metasternite of ultimate leg-bearing segment width-to-length ratio ~ 2	***M.brevisternalis* Takakuwa, 1934**
12	Trochanteroprefemur with a distal tooth, spiculum absent	***M.yanagiharai* Takakuwa, 1936**
–	Trochanteroprefemur with both basal and distal teeth, spiculum present	***M.monticolens* Chamberlin, 1920**
13	Number of leg-bearing segments invariantly 45	***M.nannocornis* Chamberlin, 1920**
–	Number of leg-bearing segments not 45	**14**
14	Number of leg-bearing segments < 60	**15**
–	Number of leg-bearing segments > 60	***M.japonicus* Meinert, 1886**
15	20 setae on each side of clypeus, sternal sulcus furcate	***M.smithii* Pocock, 1895**
–	Approx. 3 or 4 setae on each side of clypeus, sternal sulcus not furcate	***M.diversisternus* Silvestri,1919**

## ﻿Discussion

Attems (1929) proposed *Mecistocephaluspunctifrons* Newport, 1843 as the type species of the genus *Mecistocephalus*, encompassing 27 species at the time. We report two new species as belonging to the genus, *M.chuensis* sp. nov. and *M.huangi* sp. nov. Identifying new species within the genus *Mecistocephalus* is highly challenging owing to the lack of adequate illustrations and complete records; for example, images of *M.angustior* Chamberlin, 1920 are lacking and the original description is brief (only a few sentences) (Chamberlin 1920). Furthermore, descriptions of certain species (such as *M.apator* Chamberlin, 1920) often lack diagnostic information, such as the number of clypeal insulae with setae and placement of the buccal setae. In the case of the new species *M.chuensis* sp. nov. and *M.huangi* sp. nov., similarities in external morphology pose difficulties in their differentiation. The most noticeable distinction between these two species lies in the metasternite of the ultimate leg-bearing segment, with or without a pillow-like protrusion, and in the arrangement of setae on each side of the clypeus. However, these characteristics have not been consistently documented across all species and require further scrutiny to adequately characterize intraspecific variation. Therefore, redescribing poorly known species is a priority, and molecular methods might help in identifying species with insufficient descriptions.

In this study, we analyzed the phylogenetic relationships of *Mecistocephalus* in China. However, only a few *Mecistocephalus*COI sequences have been documented in the literature, in addition to several unpublished COI sequences deposited in the NCBI GenBank database (https://www.ncbi.nlm.nih.gov/genbank/). We selected twelve COI sequences from five species collected in Taiwan, including *M.guildingii*, *M.marmoratus*, *M.multidentatus*, *M.diversisternus*, and *M.japonicus*. To eliminate incorrect identifications, we also remotely examined photographs of specimens obtained from the authors who submitted the sequences (Jui-Lung Chao, pers. comm., 4^th^ Oct 2023). Phylogenetic analyses based on COI sequences from ten species with *M.rubriceps* and *M.mikado* demonstrate that *Mecistocephalus* species in China can be divided into four distinct clades. The number of leg-bearing segments in species of *Mecistocephalus* found in China can be divided into three groups: 45, 49, and > 50. Moreover, studies have shown the number of leg-bearing segments can vary within the same species (Bonato et al. 2001). In this study, neither the phylogenetic trees nor the genetic distances among *Mecistocephalus* species indicated that the evolutionary relationships are linked to the number of leg-bearing segments. This could be explained the utilization of COI sequences alone for phylogenetic reconstruction owing to inadequate 16S and 28S rRNA sequences in public databases for most *Mecistocephalus* species or could indicate that the number of leg-bearing segments does not vary within a species in China; further data are needed to evaluate this.

According to the results of the phylogenetic analysis, *M.chuensis* sp. nov. and *M.huangi* sp. nov. exhibit a close evolutionary relationship, forming a well-supported clade distinct from other species. The genetic distances between different samples of the same species were related to physical distances between localities. However, this pattern was not observed for *M.guildingii*. To improve the data set for constructing the phylogenetic tree, two additional COI sequences of *M.guildingii* (CMMI 20201214103 and CMMI 20200608031) from Yunnan Province, China were included in this study. Interestingly, the type locality of *M.guildingii* is St. Vincent and the Grenadines, a considerable distance away. Despite this, the phylogenetic analysis did not reveal a similar pattern in *M.guildingii* to those observed in *M.chuensis* sp. nov. and *M.huangi* sp. nov. The mean genetic distance of *M.guildingii* was 0.8%, compared with 5.0% for *M.chuensis* sp. nov., suggesting the possibility of cryptic species or a connection to geographic variation in *M.chuensis* sp. nov. Similarly, *M.mikado* showed an intraspecific genetic distance of 5.7%, which raises the possibility of individual variation or geographic divergence. Additionally, it should be noted that we have not examined the holotype of *M.mikado* and have classified the species based solely on the original description and illustrations, which could account for the observed genetic distance, to some extent. Moreover, a phylogenetic analysis using 78 COI sequences of *Nannarrup* did not reveal a significant correlation between the phylogenetic relationships of *Nannarrupinnuptus* samples and their geographic locations (Tsukamoto et al. 2022). Thus, the unique characteristics observed in *M.chuensis* sp. nov. and *M.huangi* sp. nov. require further confirmation with broader data. Nevertheless, genetic distance, species delimitation, and morphological identification results confirmed the taxonomic status of *M.chuensis* sp. nov. and *M.huangi* sp. nov. as new species.

The limitations of single-locus molecular data for species delimitation, the broad geographic distribution of samples, intraspecific variation, and morphological changes in individuals during development likely contributed to identification of 17 and 18 distinct units using the PTP method in this study. These constraints in species classification underscore the importance of gathering extensive genetic data across a wider range of taxa for more comprehensive analysis of the relationships within *Mecistocephalus*.

## Supplementary Material

XML Treatment for Mecistocephalus

XML Treatment for Mecistocephalus chuensis

XML Treatment for Mecistocephalus huangi

XML Treatment for Mecistocephalus smithii

## ﻿Additional information

### Author ORCIDs

Yang-Yang Pan https://orcid.org/0000-0002-8096-8550

Jia-Bo Fan https://orcid.org/0009-0006-0390-4285

Chun-Xue You https://orcid.org/0000-0002-3315-6090

Chao Jiang https://orcid.org/0000-0003-1841-1169

### Data availability

All of the data that support the findings of this study are available in the main text.
